# The influence of contextual factors on an intervention for people with disabilities from support persons’ and health personnel's perspectives: a focus group study

**DOI:** 10.3389/fresc.2024.1294990

**Published:** 2024-05-01

**Authors:** Anette Granberg, Lars-Olov Lundqvist, Anna Duberg, Marie Matérne

**Affiliations:** ^1^ Faculty of Medicine and Health, University Health Care Research Centre, Örebro University, Örebro, Sweden; ^2^ School of Behavioural, Social and Legal Sciences, Örebro University, Örebro, Sweden

**Keywords:** disability, implementation, intervention, i-PARIHS, practice

## Abstract

**Introduction:**

Contextual factors influence interventions in healthcare and pose a particular challenge in interventions designed for people with profound intellectual and multiple disabilities (PIMD). Exploring support persons’ and health personnel’s experience of an intervention may improve our understanding of the influence of contextual factors. Such exploration is important for revealing areas and focus points for future implementations. Therefore, the aim of this study is to explore support persons’ and health personnel’s experience of contextual factors during involvement in an intervention for people with PIMD.

**Methods:**

This focus group study includes eight groups, comprising a total of 34 support persons and health personnel, at habilitation centres at four regions in central Sweden. Data were analysed inductively using a content analysis approach.

**Results:**

Three themes emerged from the analysis of the informants’ perspectives on the contextual factors: (1) structure and support enhances intervention feasibility; (2) an intervention’s benefit for people with PIMD increases its acceptability; and (3) being engaged and involved increases support persons’ and health personnel’s motivation. Our findings show that the implementation of an intervention for people with PIMD should focus on the recipients of the intervention in its context, forming a clear communication plan. A training programme should be provided for the recipients and providers of the intervention.

**Discussion:**

Finally, the implementation process can be facilitated by creating space for staff to contribute and by encouraging participation and ownership for everyone involved. Using a co-design strategy can enable a shared responsibility to solve the identified challenges, while contributing to the development and design of future interventions for people with disabilities.

## 1 Introduction

Recognizing the influence of context is crucial to the implementation of interventions in healthcare (1). A large body of studies show that contextual factors—that is, all factors that are not part of the intervention itself—may act as barriers or facilitators and influence the outcome of interventions in healthcare (2–5). Consequently, we cannot understand or explain findings in this field without looking at the context in which they are embedded (1, 6, 7). The context can be characterized as the environment or setting in which the intervention is implemented, including both the physical environment and the social environment (5, 8). Among the most frequently reported factors are organizational support, financial resources, social relations, leadership, organizational culture, and climate (5). However, less is known about the influence of contextual factors on interventions in disability healthcare (9).

In healthcare for people with disabilities, the objective is to ensure that the patient maintains mobility, orientation and independence by adapting and evolving skills and functions relevant to her or his everyday life, while simultaneously considering the patient's developmental stage (10). Interventions in disability healthcare pose a particular challenge, since they frequently must be tailored to specific individual needs, have multiple outcomes and are influenced by a number of interacting contextual factors (11–14). Hence, interventions in disability healthcare are often more complex than those in other areas of healthcare (14, 15). This is particularly the case for interventions aimed at people with profound intellectual and multiple disabilities (PIMD) (16, 17). The success of interventions is influenced by the level of the rehabilitation professional involved. Variances between professionals like occupational therapists and their assistants can affect how well interventions work. Task-sharing, assigning tasks to less specialized health workers during staff shortages, is a strategy to enhance service accessibility (18)

People with PIMD have a combination of profound intellectual disability and physical impairment, along with additional sensory impairments, epilepsy or major medical health problems (19). Furthermore, people with PIMD have little or no understanding of verbal language and no apparent symbolic interaction with objects (19). These communication difficulties make people with PIMD dependent on others for all aspects of their lives (19). Since people with PIMD have extraordinary needs, they pose challenges for health personnel (20). That is, health personnel must offer a broad range of services in order to meet the specific and often complex medical and habilitation needs of people with PIMD (21). Therefore, interventions for people with PIMD can be challenging, since it is necessary to tailor the intervention to their specific individual needs, and the outcome of an intervention can be influenced by different contextual factors (14, 22). When planning for an intervention, it must be considered that people with PIMD represent a heterogeneous group with a variety of individual needs and require continuous support from support persons for a large part of their lives (23). Support persons, such as parents, relatives or others, may provide daily care to people with PIMD in their home, at the hospital or in a residential care facility. In that sense, a support person acts as the deputy, communication channel and link to society for a person with PIMD (24). Support persons are thus deeply involved when people with PIMD attend activities or receive treatments in healthcare (25). Mlenzana et al. (26) have shown that attention should be directed to health personnel to improve their interactions with support persons, because the latter are highly involved in patients’ treatment processes. However, although support persons play a central role in the habilitation process (27) and in the everyday life of people with PIMD (23), more knowledge is needed about their experiences of interventions in habilitation settings (24).

Understanding the delivery process is key in planning the future implementation of an intervention (28). In such cases, the integrated Promoting Action on Research Implementation in Health Services framework (i-PARIHS) developed by Kitson et al. (29) can be a useful tool, as it highlights the different layers of context for successful implementation (30). The i-PARIHS framework builds on four constructs for successful implementation: *facilitation*, *innovation*, *recipients* and *context*. Facilitation is the active element that assesses, aligns with and integrates the other three constructs (30, 31). Each of the constructs includes a number of characteristics to be considered for implementation (30). *Innovation* considers the characteristic of knowledge and how it affects the practical application of a new intervention; *recipients* considers the impact of individuals or groups in supporting or resisting innovation; and *contexts* includes the resources, culture, leadership, policy and capacity for innovations to be implemented at different levels, such as the local, organizational and wider healthcare system. The results from this study will be discussed in light of the i-PARIHS framework to reveal areas and focus points for the future implementation of interventions for people with PIMD.

Since health personnel and support persons play a central—albeit possibly different—role in interventions, exploring their experience of contextual factors in an intervention will improve our understanding and facilitation of future interventions and their implementation.

Therefore, the aim of this study is to explore support persons’ and health personnel’s experience of contextual factors during their involvement in an intervention.

## 2 Methods

### 2.1 Settings

This study is part of a multicentre randomized crossover study investigating the effects of the Structural Water Dance intervention (SWAN) for people with PIMD (32). The goal of the SWAN program is to enhance the well-being of individuals with PIMD. It aims to achieve this by alleviating stress, spasticity, and pain, while also enhancing alertness, overall well-being, quality of life, and social interaction. SWAN was performed at adult habilitation centres in four regions in central Sweden. The intervention was conducted as a group activity with four or five patients in a warm pool, led by two health personnel (physiotherapists, assistant physiotherapists, or pedagogues) and held once a week for three months (12 sessions in total) (32). The intervention is primarily aimed at patients. However, considering the significant disabilities of these individuals, assistants play a vital role in its implementation. The effectiveness of the intervention for participants with PIMD greatly depends on the involvement and support of these assistants. Each individual with PIMD received support from two support persons (i.e., residential care personnel, a personal assistant, or a parent), who accompanied each individual with PIMD and functioned as a dance partner in the water. Prior to the intervention, the health personnel received comprehensive training in the theory, method, and practical aspects of the SWAN program. This training equipped them to guide and support the assistants in their role as dance partners. Additionally, the research team made regular visits to each participating center. These visits were crucial for conducting measurements and addressing any issues that may have emerged. Hence, the SWAN intervention offered an opportunity to investigate in more detail health personnel's and support persons’ experience during an intervention.

### 2.2 Characteristics of the informants

We considered it essential to obtain informants’ experiences of the SWAN intervention from different perspectives. Therefore, we invited all 43 potential informants (36 support persons and seven health personnel) to participate in the focus groups. Of those invited, 34 participated (27 support persons and seven health personnel), including 32 women and two men, with a mean age of 44 years. Some declined due to a high workload, and some of those invited did not respond to the invitation. The number of support persons and health personnel ranged from six to 13 per region. Details of the informants are provided in Table 1. The informants were a mix of residential care personnel, personal assistants, parents, physiotherapists, assistant physiotherapists and pedagogues. The informants were informed of the purpose of the study, assured confidentiality, and reminded that they were free to terminate their participation in the study at any time. All informants gave written informed consent before the focus group started.

**Table 1:** Details of the informants; support persons and health personnel.

	Support persons	Health personnel	Total
Gender			
Women	25	7	32
Men	2	0	2
Age			
Range	21–64 years old	35–63 years old	
Mean	41 years old	55 years old	
Organization			
Regional sector	0	7	7
Municipal sector	15	0	15
Private sector	12	0	12
Education			
Lower secondary school	2	0	2
Upper secondary school	22	3	25
Post-secondary education or vocational training	1	0	1
University	2	4	6
Professions/role			
Residential care personnel	6	0	6
Personal assistant	18	0	18
Parent	3	0	3
Physiotherapist	0	2	2
Assistant physiotherapist	0	3	3
Pedagogue	0	2	2
Years of experience			
Range	2–30 years	1–45 years	
Mean	13 years	24 years	

### 2.3 The focus group

We conducted eight focus groups between February 2019 and February 2020. Seven focus groups were conducted with support persons and one was conducted with health personnel. The groups were homogeneous, based on the informants’ professions and functions, since it can be assumed that participants with common experiences and areas of interest will be more willing to share their opinions with each other (33). Our goal was to have at least three support persons in each of the support person groups; however (due to sick leave and reprioritization), two focus groups had only two support persons each. All seven health personnel were placed in a single focus group that included one participant from one region and two from each of the three remaining participating regions.

### 2.4 Data collection

When developing the interview guide, we used the i-PARIHS framework as an inspiration (30). The questions focussed on contextual factors and their potential impact, based on participants’ experiences of the SWAN intervention; more specifically, they focussed on the intervention, informants’ motivation, skills and knowledge, the environment and the collaboration (see Supplementary Material for interview guide). The questions were sent to the informants 1 week before the focus group session to allow them to prepare for the focus group (33). At the beginning of each focus group, the participants were informed about the purpose of the study and the rules of conduct, including the need to show respect for the other participants. A research team member (AG) moderated and initiated the discussions and focussed on the interactions between informants to ensure that there was enough space for individual perspectives and informants’ views to be generated. We also ensured that there was an opportunity to explore the unforeseen areas that came up in the interactions between the informants. An observer (MM) documented the conversation, the speech order and the interaction to make sure that all the informants had enough space to talk. The focus groups were audio-recorded, and the length of the focus groups varied from 75 to 90 min. The focus group recordings were transcribed verbatim and stored in data files that were only accessible to authorized persons.

### 2.5 Data analysis

We analysed the data using a qualitative content analysis with an inductive approach, guided by the step-by-step process described by Graneheim and Lundman (34). All the authors (AG, AD, LOL and MM) were engaged in the initial discussion on how the data would be interpreted. Units of meaning corresponding to the study aim were selected first. Next, the analysis process focussed on the content and contextual meaning of the informants’ statements. The statements were condensed while retaining information on what each statement was about. The condensed units were then formed into codes. When the authors’ opinions regarding the codes differed, AG and MM discussed and modified the codes until consensus was reached. Thereafter, AG and MM independently grouped the codes under higher headings through the generation of subcategories and categories. These categories illustrated the level of the content and expressed the manifest content of the text. Based on the categories, all the authors (AG, AD, LOL and MM) collaborated to develop themes that explained the underlying, latent meaning throughout the material. These themes were then discussed with all the authors until consensus was reached. NVivo 12 software was used to organize, store and analyse the data (35). The Standards for Reporting Qualitative Research (SRQR) were used during the manuscript preparation (36).

## 3 Results

Three themes emerged from the analysis of the influence of contextual factors on the SWAN intervention from the informants’ perspectives: (1) support and structure enhances intervention feasibility; (2) an intervention's benefit for people with PIMD increases its acceptability; and (3) being engaged and involved increases support persons’ and health personnel's motivation. Table 2 provides an overview of the themes and categories.

**Table 2:** Themes and categories.

Themes	Categories
1. Support and structure enhances intervention feasibility	1. Support and resources 2. Practical dilemmas 3. Communication and training
2. An intervention's benefit for people with PIMD increases its acceptability	1. Value of the method 2. Joy of movement 3. Positive interaction
3. Being engaged and involved increases support persons’ and health personnel's motivation	1. Learning environment 2. Continuity and collaboration 3. Ability to lead

Under each theme described below, the categories are exemplified with quotations. The two types of informants are referred to as ’support persons’ or ‘health personnel’, while the term ‘informants’ is used to collectively refer to all participants in the focus groups.

### 3.1 Theme 1: support and structure enhances intervention feasibility

The first category in this theme involves having *support and resources* to participate in the intervention, such as committed managers. Certain residential care personnel and personal assistants (support persons) reported that challenges such as high staff turnover and declining economic conditions necessitated their participation in the intervention sessions during their free time. Furthermore, the health personnel were concerned that the support persons could have very different expectations for the time required to participate in the session. Despite these challenges, there was a consensus that the intervention had potential to improve the well-being of individuals with PIMD's, underscoring the continued participation in the intervention sessions.

*Our manager thinks it is very positive that we are involved in this, and she has been checking on how it has been going. (Support person focus group 1)*

*There will be extra staff, and that is quite a large expense, and we have been given an hour to share, and it has to do with finances, and I hope that we will have the resources to continue. (Support person focus group 3)*

*The crucial thing is that there are so many assistant companies (…) because it’s very strange that some solve it [companies ensuring that employees have sufficient time to participate in the intervention) and (…) while others claim that it’s impossible. It should be the same situation, one might think, but they are different companies. (Health personnel focus group 5)*

*The manager has been positive about him [a person with PIMD] going to the SWAN sessions, and it is by far his most enjoyable and best activity. (Support person focus group 3)*

The second category, *practical dilemmas* involves circumstances or situations that the informants perceived as things that they could not control, and the extra work the SWAN sessions entailed. Examples included waiting for transport and/or long travel times to and from the intervention sessions. Some support persons felt that they could not relax and were constantly prepared during the transport for any events that could negatively affect the person with PIMD in any way. This was perceived as being stressful for the support persons.

*There is a lot of work with the logistics before we get into the water, regardless of whether it is waiting for taxis, changing rooms and lifts. (Health personnel focus group 5)*

*To go from the shower bed over to the water was a very difficult moment for us and, after three times, I had to go and talk to them. The move itself was difficult and a stressful moment, they had no idea how it was going to happen. The wait can be stressful in itself, you have to wait for a lift and wait and wait. (Support person focus group 4)*

Finally, the third category within this theme is *communication and training*, which describes how the informants perceived the information and anchoring of the intervention, and how the intervention was organized. The support persons expressed that they wished they had been more prepared for what would happen during the intervention. They lacked a structure for the intervention sessions and were concerned that they had not received any training before the intervention started.

*It would have been good if all the support persons had received the same training as the health personnel, because they might have felt more engaged in the project if they had had the same training, because people feel more secure when they understand what is going on. (Support person focus group 8)*

### 3.2 Theme 2: An intervention's benefit for people with PIMD increases its acceptability

The first category, *value of the method*, involves the structure of the intervention sessions. In the SWAN intervention, the order of various elements was repeated in the same way at each session throughout the whole intervention period. The dance movement combinations and music were perceived as being well planned and having good variation for people with PIMD. The intervention's balance between slow exercises and movements (e.g., stretching muscles) and fast-paced music that raised the heart rate was something that the informants expressed that their people with PIMD had not encountered much or had not done before. The informants appreciated that their person with PIMD could do the sessions together with others, because this heightened the experience for the people with PIMD, according to the informants. The informants praised the intervention method's focus on the *joy of movement* (the second category), which they viewed as new and exciting, and several informants perceived the method as being highly relevant for people with PIMD. The support persons explicitly expressed the importance of offering meaningful activities—like the current intervention method—in comparison with those the support persons perceived to be substandard and not prioritized for this group.

*I mean, you can enjoy other activities too, but, but this was something special. It was so different from what we had done before, and I thought it was exciting as well as fun. (Support person focus group 8)*

*I think it was the fact that it was not a matter of training, but just an easy-going, playful programme. No focus on any particular illness (…). (Support person focus group 4)*

Most of the informants were willing to recommend the intervention to other patient groups in a similar situation. However, some health personnel expressed concern about what people from within their organization would offer the intervention, stating that they themselves did not have sufficient time, and discussing where they thought the responsibility lay. Some informants saw opportunities to offer the intervention with minor adjustments, and some support persons expressed that the intervention suited their person with PIMD much better than other activities offered by the habilitation centres. The support persons expressed that further value was added to the method by the *positive interaction* (the third category) between the support person and the person with PIMD, as they did things together while participating in the intervention sessions. For example, the person with PIMD would come up in a standing position in the water and dance with the support person during one song in the SWAN session.

*Amazing when you got up, and he probably felt like a prince, and we felt like the princess. Because it was absolutely incredible, that smile, and he is not an expressive guy, but so happy (…) you want to cry because it was so awesome. (Support person focus group 2)*

### 3.3 Theme 3: being engaged and involved increases support persons’ and health personnel's motivation

In this theme, the first category is the *learning environment*. The support persons expressed that they experienced an environment that was flexible and open to learning during the intervention, which they considered had a positive effect on their own development and commitment to engaging in the intervention. Some supporters expressed that they received many ideas about how to make water exercise more enjoyable for their person with PIMD. Others expressed that they liked being prepared for each session. By getting involved and actively learning the exercises and dances, they could become a better support for their person with PIMD, resulting in a better experience for the person with PIMD in the end.

*I get to be a part of it. I have evolved, particularly through what we do together. (Support person focus group 3)*

*It is a boost, particularly for us support person, to interact with others and do things together (…). I think it is very important to meet others, compare notes, and find out what you really know. (Support person focus group 6)*

Some health personnel expressed that the extent to which the support persons were involved could affect the person with PIMD’s experience of the intervention.

*(…) a few of them had…an amazing support person one time, and then a different one another time, one who neither had any idea of pacing nor understood what we were doing (…) And what’s that like for the person, I wonder? Therefore, it is very important; in fact, it is essential, to have the right people in the right place. (Health personnel focus group 5)*

The second category is *continuity and collaboration*. The health personnel experienced the benefit of people with PIMD having the same support person throughout the intervention, which they felt facilitated the collaboration between the support persons and the health personnel. When the support persons knew the procedures, they knew what was expected of them during the intervention session, and they understood how to deal with any problems that arose that might affect their person with PIMD during the session. The health personnel expressed that they were generally confident in their role and mission; they participated actively and were engaged in the task. The health personnel felt that they worked together for a common purpose, for the benefit of people with PIMD. Furthermore, the health personnel expressed and emphasized the continuity of having the same support persons who knew the intervention programme, because it made for a better experience for the people with PIMD.

*(…) it shows in the results—those who have the same support person every time have learned how to perform the movements and can do them whether they are in the water or next to it. And this usually translates into an utterly wonderful result for the patient. (Health personnel focus group 5)*

The third category is the *ability to lead*. Despite support from the health personnel, some support persons expressed that the sessions could be perceived as stressful, because the support persons were unsure about their own role and function in that specific situation. Some health personnel had the ability to lead and experienced the leading as simple, whereas others expressed having difficulty communicating the purpose of the intervention and motivating the support persons to be actively involved in the intervention sessions. Some support persons felt safe and knew what to do because of the support from the health personnel during the sessions.

*I explained: ‘Excuse me, but this is my first time, so I have no idea what to do’. Then there was someone in the pool: ‘No, but it’s easy, I'll help and show you’. (Support person focus group 7)*

## 4 Discussion

### 4.1 Discussion in relation to the results

The aim of the present study was to explore support persons’ and health personnel's experience of contextual factors during involvement in an intervention. The findings emphasize that leadership and management support, such as engagement and feedback can facilitate the feasibility of the SWAN intervention. The support persons and health personnel expressed barriers related to communication and training, such as support and resources, and practical dilemmas. This finding aligns with recent research highlighting the importance of supportive components such as education and training, marketing and awareness, and auditing and feedback (37, 38). In addition, other studies have noted that support persons’ commitment to and support for an intervention affect the success of its implementation (39, 40). To increase commitment and support for the intervention, clear communication about the purpose of the intervention and a focus on the goal of the intervention may improve the intervention's clarity. Research highlights the importance of the involvement of ‘the end users’ (27), such as healthcare and social care professionals, who may utilize the research findings in their daily practice. Support persons can play a central role in the organization and facilitate the implementation of the SWAN intervention, as they can advocate for the needs of people with PIMD (27).

The informants expressed that the intervention was perceived as appropriate, relevant and acceptable as an intervention for people with PIMD. Research emphasizes that interventions should be adjusted in a way that is suitable to the local setting (37, 41). The health personnel foresaw barriers in possibly adapting the intervention to local settings, such as a lack of time to carry out the intervention and the question of which persons within the organization should be responsible for the continuation of the SWAN intervention. These aspects may require further investigation, since studies have shown that the implementation of new interventions in hospital settings is extremely challenging, even when new interventions are widely accepted (37).

Our findings show that the health personnel expressed the importance of continuity—that is, having the same support person be present at each intervention session—not only for benefiting the person with PIMD but also for facilitating the cooperation between the support persons and health personnel. Thus, the support person may in a sense have two complex roles in such an intervention, being both a representative of the person with PIMD and a support person with wider duties and responsibilities. Recent research emphasizes the need for clarity regarding participants’ roles—both for health personnel and within teams—in order to achieve effective interventions (26, 27). The support persons experienced barriers associated with the intervention, which may explain why cooperation can be difficult between support persons and health personnel due to the diverse focus of the two staff groups—that is, the support persons’ focus was on caring for the person with PIMD, while the health personnel's focus was on delivering the SWAN sessions. To increase involvement in and motivation for the SWAN intervention, a co-design approach can be used, which can enable support persons and health personnel to contribute to finding solutions for identified barriers. This approach goes beyond consultation by building and deepening an equal collaboration between the support persons and health personnel; these users, as ‘experts’ in their own experience, can be central to facilitate the implementation process for an intervention (42).

### 4.2 The i-PARIHS framework in relation to the results

The i-PARIHS framework emphasizes the importance of considering the relation between the constructs of innovation, context (micro, meso, macro), recipients, facilitation process, and a number of other characteristics, in order to enable a successful implementation (30) (see Table 3). The barriers identified by the support persons in this study can be associated with the recipients’ constructs within the i-PARIHS framework (i.e., the impact of the staff supporting or resisting an intervention), such as time, resources, support, collaboration and teamwork, and skills and knowledge, in participating in and delivering the SWAN intervention (see Table 3). Furthermore, barriers can be associated with the context constructs i.e., different layers of context within the local (micro), organizational (meso) and wider health system (macro), such as culture, which are involved in decisions on how to organize and structure the SWAN sessions for persons with PIMD. Furthermore, access to other interventions for persons with PIMD—where such interventions were described by the support persons as infrequent and substandard—may affect how organizations prioritize interventions and could be associated with context constructs at the organizational level (meso) and organizational priorities within the i-PARIHS framework. To facilitate future implementation of the intervention, strategies can be developed to address and manage these barriers.

**Table 3:** Themes and categories within the context of the i-PARIHS framework constructs and characteristics.

Themes	Categories	Interpretation of facilitators/barriers	The i-PARIHS framework within the constructs: innovation, recipients and context (micro, meso, macro), and characteristics
1. Support and structure enhances intervention feasibility	1. Support and resources *(managers’ engagement and support, resources)*	Barriers Facilitators	**Recipients:** Time, resources, support *(necessary time, resources, support)* **Context (meso):** Senior leadership and management support *(engagement and regular feedback)*
	2. Practical dilemmas *(lack of control of the situation)*	Barriers	**Recipients:** Time, resources, support *(support)*
	3. Communication and training *(the purpose of the intervention, involving support persons in decisions, providing training)*	Barriers	**Recipients:** Motivation *(supporting or resisting the innovation)* Goals *(clear and accepted goals)* Skills and knowledge *(necessary skills and knowledge)* **Context (micro):** Culture *(one that supports innovation, and staff being involved in decisions that affect them)*
2. An intervention's benefit for people with PIMD increases its acceptability	1. Value of the method *(design of the method, well planned, access to guidelines)*	Facilitators	**Innovation:** Usability *(tool availability)*
	2. Joy of movement *(the intervention was appreciated by the informants, relevant for people with PIMD and viewed as a meaningful activity)* *(little and substandard access to intervention)*	Facilitators Barrier	**Innovation:** Relative advantage *(the degree to which an innovation is seen as better than the idea, programme or product it replaces)* Trialability *(the potential to test the intervention on a small scale)* Observable results *(observable benefits and improved outcomes)* **Context (meso):** Organizational priorities *(the innovation aligns with organizational priorities)*
	3.Positive interaction *(between the support persons and people with PIMD added further value)*	Facilitators	**Innovation:** Observable results *(observable benefits and improved outcomes)*
3. Being engaged and involved increases support persons’ and health personnel's motivation	1. Learning environment *(being together within a context)*	Facilitators	**Recipients:** Collaboration and teamwork *(boundaries and relationship between groups or teams)* **Context (micro):** Learning environment *(fostering a learning environment)*
	2. Continuity and collaboration *(action between receiving and delivering, continuity)*	Barriers	**Recipients:** Motivation *(supporting or resisting the innovation)* Collaboration and teamwork *(boundaries and relationship between groups or teams)*
	3. Ability to lead *(skills, knowledge, role and function)*	Facilitators or barriers	**Recipients:** Skills and knowledge *(necessary skills and knowledge)*

Characteristics that can be considered within the context of the i-PARIHS framework Innovation constructs: Underlying knowledge sources, Degree of fit with existing practice and values (compatibility or contestability).

Recipients’ constructs: Values and beliefs, Local opinion leaders, Existing networks, Power and authority, Presence of boundaries.

Context constructs: Micro level—Past experience of innovation and change, Evaluation and feedback processes; Meso level—Culture, History of innovation and change, Absorptive capacity; Macro level—Policy drivers and priorities, Incentives and mandates, Regulatory frameworks, Environmental (in)stability, Interorganizational networks and relationships (30).

Enabling factors for the SWAN intervention can also be associated with context constructs (meso) within the i-PARIHS framework, such as engaged and committed managers, and with innovation constructs, such as the utility, benefit, testability and observable outcomes of the SWAN intervention for people with PIMD. However, even though the intervention seemed to be widely accepted, it might be necessary to consider additional factors affecting implementation within the context construct. For example, at the local level (micro), unit, department or team and at the organizational level (meso), unit, department or team embedded in the organization), such factors might include the degree of knowledge of past experiences of innovations and change, evaluation and feedback processes, culture, and the ability of organizations to absorb a new intervention. Furthermore, the wider healthcare system (macro) in which the organization is based, as well as the policy, social, regulatory and political infrastructures surrounding the local context, are factors that could possibly facilitate or hamper the implementation of the SWAN intervention (30). Other possible factors to consider were not as prominent in our results and can therefore be examined more closely in future implementations of the intervention. For example, within the innovation constructs, the factor of underlying knowledge sources relates to knowledge that may affect the SWAN intervention's migration and uptake in different settings, as well as the degree to which the SWAN intervention aligns with existing practices and values (i.e., compatibility or contestability). Furthermore, within the recipients’ constructs, other factors could include the support persons involved in the implementation, including their values and beliefs, local opinion leaders, existing networks and established ways of practice—which can present challenges affecting the ease of introducing the SWAN intervention.

In addition, the facilitation construct (which refers to the process of supporting the implementation of innovation within the i-PARIHS framework) was not commented by the informants in the results. This result was perhaps not surprising, as the purpose of this study was not to implement the SWAN intervention but to explore support persons’ and health personnel's experience of contextual factors during their involvement in the intervention. However, since facilitation is an essential construct for successful implementation within i-PARIHS, exploring the opportunity for a facilitation process may be beneficial for future implementation of the SWAN intervention. Thus, the facilitation process or facilitator's role plays a particularly central role in overcoming identified barriers for implementation (43).

## 5 Strengths and limitations

This study has some limitations. As the informants knew that the researchers hosting the focus groups were part of the team behind the intervention, there is a risk that they praised the intervention as a way of showing their gratitude for any beneficial results. However, the informants expressed both negative and positive responses about their experience in the intervention.

AG and MM sent out the questions in advance so the informants would know and feel comfortable with the questions to be addressed in the focus group discussions. Nevertheless, this advance knowledge may have been a disadvantage, as it may have limited the informants’ openness to the discussion. MM and AG tried to create a thoughtful, open atmosphere by providing an overview of the topic and ground rules to set the tone of the discussion. They also tried to encourage the participants to share their point of view, and emphasized that the researchers were equally interested in negative and positive comments.

As another possible limitation, the focus groups may not have reached the maximum possible depth on particular issues, in comparison with individual interviews, and the informants may not have expressed their honest and personal opinions about the topics due to having multiple listeners. However, the focus groups enabled the researchers to gain insight into a wide range of attitudes, knowledge and experiences, because we provided the opportunity to explore the participants’ experiences that came up in the interactions between informants. To increase the credibility of the data collection, two authors (MM, AG) followed the entire process and worked together throughout the analysis process, which strengthened the trustworthiness of this study. This study highlights challenges regarding support persons’ and health personnel's experience of contextual factors during their involvement in an intervention for people with PIMD; therefore, the findings may not be transferable to other healthcare settings.

## 6 Conclusion

We concluded that an equal and active collaboration between the support persons and health personnel in the SWAN intervention enhanced the feasibility and increased the acceptability of the intervention. Support persons’ and health personnel's motivation was improved if they considered the intervention to be beneficial to its intended targets. To overcome the identified barriers, attention should be focused on forming a clear communication plan, carrying out a training programme for the recipients and providers of the intervention, and enabling a facilitation process by creating space for staff to contribute and by encouraging participation and ownership for all involved. Using a co-design strategy can enable a sense of shared responsibility in solving the identified barriers and can also contribute to the development and design of future interventions for people with disabilities. Although different contexts may have different prerequisites, the results of this study can guide future planning for the implementation of interventions within habilitation settings for people with disabilities.

## Data Availability

The original contributions presented in the study are included in the article/**Supplementary Material**, further inquiries can be directed to the corresponding author.
