# Supplementary material for: The influence of contextual factors on an intervention for people with disabilities from support persons’ and health personnel's perspectives: a focus group study
Source: Front Rehabil Sci. 2024 May 1;5:1294990. doi: 10.3389/fresc.2024.1294990 (PMC11094220; doi:10.3389/fresc.2024.1294990)
Supplement: Supplementary file 1 [file Table1.docx]

**Additional file 1 – Interview guide**

| **Characteristics** | **Supporting questions and descriptions** | **Interview questions** |
| --- | --- | --- |
| **Context** | What enables and hinders the implementation of the intervention? What processes and networks are central for the implementation of the intervention?  How the intervention was performed in practice. Everything that the participants were involved in, such as getting to the activity, changing rooms, showers and other practical things in connection with the intervention. Furthermore, how the participants perceived the information, communication and introduction of the intervention. The processes before, during and after the intervention started, and the resources needed to participate. | 1. Describe your experience with the intervention. What worked well? What worked less well? |
| **Innovation** | The method itself (the need for the method) and its adaptability to the habilitation setting.  The experience of the programme (movements, dance to music) within the intervention. | 2. What is your experience of the intervention? |
|  | How did the patient express their participation during the intervention? What did the patient express and in what way? | 3. How did you perceive the patient’s experience of the intervention? |
| **Recipients** | What is essential for the quality of the work?  What is central to succeed in the role/tasks?  What is key for collaboration/cooperation?  In what way do you learn from each other? | 4. How did you experience your role during the intervention session? (as a companion in the pool or next to it) |
|  | What is essential to consider for motivation?  What is important for motivation regarding the group/team? What is central for you to have an influence over participation in the intervention? | 5. How do you see your commitment and motivation to participate in the intervention, and in what way does it affect you? |
|  | What enables the implementation of the intervention? | 6. What do you think is most essential to highlight about implementing the intervention? |
|  | Will the experience of the intervention change your way of working? If so, in what way? | 7. How do you see the continuation of the intervention in habilitation settings? |
|  | Support from managers | 8. Have your managers commented on your participation in the intervention? |
| **Closing question** |  | 9. Is there anything that you want to add? |
